# Bacterial ghost of avian pathogenic *E*. *coli* (APEC) serotype O78:K80 as a homologous vaccine against avian colibacillosis

**DOI:** 10.1371/journal.pone.0194888

**Published:** 2018-03-22

**Authors:** Hakimeh Ebrahimi-Nik, Mohammad Reza Bassami, Mehrdad Mohri, Mehrnaz Rad, Mazhar I. Khan

**Affiliations:** 1 Department of Pathobiology, Faculty of Veterinary Medicine, Ferdowsi University of Mashhad, Mashhad, Iran; 2 Department of Clinical Sciences, Faculty of Veterinary Medicine, Ferdowsi University of Mashhad, Mashhad, Iran; 3 Department of Pathobiology and Veterinary Science, University of Connecticut, Storrs, CT, United States of America; Instituto Butantan, BRAZIL

## Abstract

Avian Colibacillosis is among the major causes of economic loss in the poultry industry worldwide, with a more vivid impact on developing countries. The involvement of several bacteria has made it challenging to develop effective vaccines for this disease, particularly because it is notoriously difficult to make a vaccine that contains all the contributing pathogenic bacteria. Here, we report the design and fabrication of a bacterial ghost (BG) of *E*. *coli* O78:K80, which is among the major bacterial serotypes responsible for this disease. The generated ghost is then exploited as a homologous vaccine against Avian Colibacillosis. We demonstrate that hole formation in the cell wall of *E*. *coli* O78:K80 can happen properly in optical densities as high as 0.8 compared to the 0.3–0.4 standard for bacteria like *E*. *coli* TOP10. This is especially advantageous for mass production of this ghost which is a vital factor in development of any BG-based vaccine. Compared to *E*. *coli* TOP10, we faced a great challenge in transforming the wild type bacteria with the E-lysis plasmid which was probably due to higher thickness of the cell wall in O78:K80. This, however, was addressed by treating the cell wall with a different combination of ions.The vaccine was administered to Ross 308 broiler chickens via injection as well as through their respiratory system at a dose of 1010 BGs, repeated 3 times at weekly intervals. Chickens were then challenged with the wild type O78:K80 at a dose of 1011 bacteria together with Infectious Bronchitis H120 vaccine (as immunosuppressant) one week after the last immunization. Air sac lesions were significantly reduced in BG vaccinated groups in comparison with the control group. The levels of IFN*γ*, IgA and IgY were measured in the serum of immunized chickens as an indication of immune response and were compared with those of the chickens vaccinated with killed bacteria. The results show that O78:K80 BG can be used as an efficient homologous vaccine against Colibacillosis disease in poultry. We expect our findings can serve as the starting point for designing more sophisticated vaccines that contain all three major pathogenic bacteria involved in avian Colibacillosis.

## Introduction

The global poultry industry loses millions of dollars every year because of Colibacillosis. Caused by infection with Avian Pathogenic *Escherichia Coli* (APEC), Colibacillosis is among the most morbid and mortal of poultry bacterial infections [1], which in turn leads to significant reduction in the production of poultry meat and eggs [2]. The disease appears in different forms from acute (septicemia) to sub-acute including pericarditis, perihepatitis, arthritis, airsacculitis and cellulitis [3].

Colibacillosis oftentimes is a secondary bacterial infection predisposed by deficient biosecurity practices as well as viral infections such as bronchitis. Typical treatment strategies include rigorous control of predisposing factors, administration of antibiotics in the primary stages of the disease as well as vaccination. Unfortunately, excessive usage of antibiotics has promoted formation of antibiotic-resistant serotypes of *E*. *coli* in poultry [4, 5], leading to less effective treatment of Colibacillosis using this method. Further, from the perspective of human health, it is favorable to minimize usage of antibiotics in food animals including poultry [6]. Three types of *E*. *coli*, namely O78:K80, O2:K21 and O1, mostly, contribute to the formation of the disease [7]. The fact that, multiple bacteria are involved, poses a huge problem in designing an effective vaccine for this disease. In fact, a sufficiently effective vaccine should be multivalent, i.e. it should be able to protect against more than one type of *E*. *coli*. Nevertheless, current commercially available vaccines fail to deliver full protection against all serotypes engaged in Colibacillosis.

Fortunately, the advent of bacterial ghost (BG) technology has opened up new avenues for developing vaccines against gram negative bacterial infections. BGs are the result of discharging all the cytoplasmic content of the gram negative bacteria, leaving behind an empty, yet intact, cell wall by controlled expression of the E-lysis gene of bacteriophage PhiX147 inside the bacteria [8]. The expressed E-lysis gene encodes a 91-amino acid protein inside the bacteria, its oligomerization capability [9] then forms a transmembrane tunnel structure in the cell wall, fusing the inner and outer bacterial cell membranes [10]. This process results in formation of pores on the cell wall that are mostly populated at polar sites or at the center of the bacteria. The cell content is then discharged, following several washing steps through the generated pores.

Two unique features of the BGs make them very attractive for vaccine development: (1) Since BGs are not alive, proliferation does not occur. Thus, they provide a safer solution relative to live vaccines. (2) The fact that no inactivation procedure is required to make BGs, all relevant immunogenic determinants on the cell wall remain intact in such a way that they can mimic certain properties of live bacteria such as surface pathogenic associated molecular patterns (PAMP) [11]. More so, BGs are shown to be capable of simulating both humoral and cellular arms of immune responses, which is particularly advantageous for developing a vaccine against Colibacillosis as it has been shown that both humoral and cellular immune responses are required to battle Colibacillosis [12].

This paper presents the design and development of a BG for APEC O78:K80 to evaluate the efficacy of this method for vaccine development against Colibacillosis. This was accomplished by exploiting E-lysis gene expression as well as recombinant technology via pmET32b which, was engineered by altering pmET32a (accession number JX518291), as a part of this work [13]. Using pmET32b was particularly advantageous because its promoter, *γ*PR, is inducible at higher temperatures while it yields minimal leakage at lower temperatures. More elaborately, at temperatures below 30°, the inhibitor CI857, a mutant protein, is capable of attaching to the promoter to inhibit the attachment of RNA polymerase and consequently blocking the transcription, however, due to denaturation and loss of 3D structure at temperatures above 30°*C*, this process does not occur. It has been observed that 42°*C* is the optimum temperature for CI857 denaturation and consequently for E-lysis gene expression.

Also, CFU determination and scanning electron microscopy (SEM) imaging were performed which confirmed the formation of BGs. Furthermore, it was observed that hole formation for *E*. *coli* O78:K80 could happen properly in optical densities as high as 0.8–0.9 compared to the 0.3–0.4 standard for bacteria like *E*. *coli* TOP10 which is beneficial for mass production of the vaccine. *In vivo* studies conducted to evaluate the immunogenicity of O78:K80 bacterial ghost, as a homologous vaccine against avian Colibacillosis, yielded reduced air sac lesions in immunized chickens versus the control group and the measured levels of interferon gamma (IFN*γ*), IgA and IgY in the serum of BG-immunized chickens were comparable to (or higher than) the chickens vaccinated with killed bacteria plus adjuvant.

**Fig 1:**
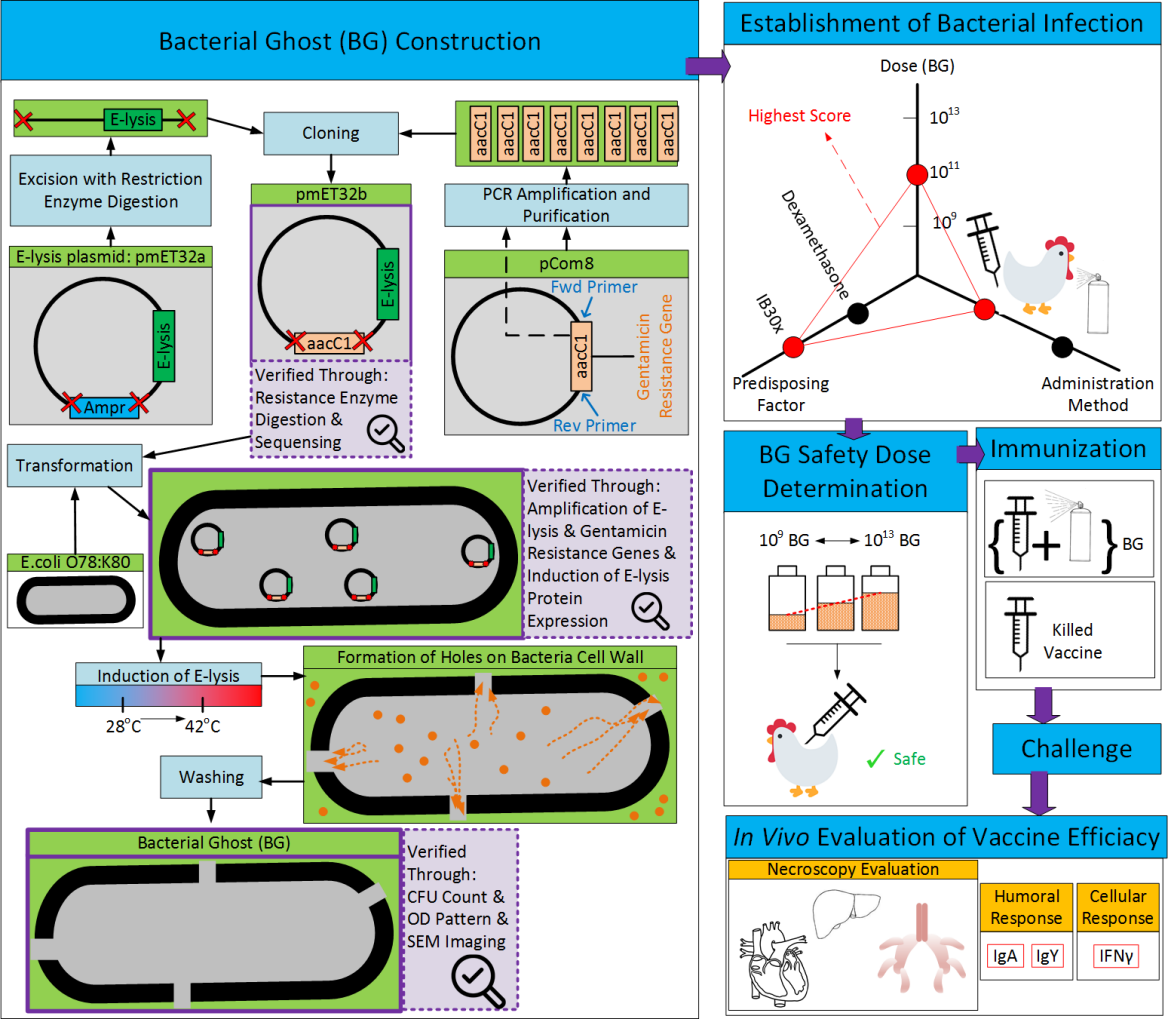
Schematic summary of production process for O78:K80 BG vaccine and it’s *in vivo* efficacy evaluation.

The developed product can be used not only as an effective vaccine against one of the main serotypes of avian Colibacillosis, but also sets the ground for designing multivalent as well as autogenous vaccines that can potentially have higher efficacies. A schematic summary of the work can be seen in Fig 1.

## Materials and methods

The vaccine production process as well as its *in vivo* evaluation will be elaborated in this section. All animal studies are conducted according to the standards practiced in Veterinary Medicine School of Ferdowsi University of Mashhad, which are in keeping with world class standards and protocols [14, 15]. All animal studies were reviewed and approved by a veterinary committee consisting of veterinarians, expert in various fields (ethics, veterinary clinical studies and etc.) in Veterinary Medicine School of Ferdowsi University of Mashhad, under the protocol number of “25893”. Briefly, birds were sacrificed when any signs of discomfort were observed. As recommended by [15], decapitation was used with dedicated and clean equipment (sharpened large blade knife which in one quick motion remove the chicken’s head completely) and it was ensured that the head is separated from the body completely and quickly by a person with technical proficiency. Also, as recommended by the guidelines in [14], non-SPF Ross 308 broiler chickens (Simorgh chicken farm, Khorasan Razavi, Iran) were maintained under standard lighting, feeding and vaccination program prescribed for broiler chickens in pens with wood shavings (chicken farm of Veterinary Medicine School, Ferdowsi University of Mashhad, protocol number: 25893).

### Bacterial strains

Wild type O78:K80 bacteria were generously gifted to us from the University of Tehran [16, 17]. As described by the authors, virulent *E*. *coli* O78:K80 was isolated from chicken with systemic Colibacillosis. Multiple biochemical tests were conducted in their study to assess the biochemical properties of the isolated bacteria [16, 17]. These tests consisted of lactose fermentation, Methyl Red and Voges-Proskauer (MRVP), citrate test and indole test and were performed using the following culture media: TSI, MRVP, Simmons citrate and peptone water, respectively. Moreover, the bacterial serotype was identified as O78:K80, using MAST serotyping kit; MAST Group Ltd, Merseyside, UK. Also, chicken embryo lethality test was utilized to assess the virulence of the bacterial strain [16]. *E*. *coli DH*5*α* was purchased from Invitrogen.

### Antibiogram test

Using a sterile glass spreader, 100*μl* of the standardized bacterial suspension (i.e., a suspension with a turbidity corresponding to 1.0 McFarland standard) was spread on an agar plate. Antibiotic disks (“Ampicillin 10”, “Chloramphenicol 30”, “kanamycin 30”, “sulfametoxazol 100”, “Gentamicin 10” and “Neomycin 30”) were located on the plate after 15 minutes. The plate was read after 24 hours.

### pmET32b vector construction

Plasmid pmET32b, a derivative of the plasmid pmET32a, was constructed by replacing the *β*-lactamase gene with *aacC1* gene.

### Gentamicin resistance gene (*aacC1*) amplification and purification

Forward and reverse primers with sequences TTGGTCATGAGATGGAAGCTAGAGTAAGTAGT and TTGGTCATGAGATGAGGCGAATTGACATAAG (both having BspHI restriction enzyme site at the 5’ end) were used to amplify *aacC1* gene, its promoter as well as its terminator from plasmid pCOM8, with accession number of AJ299427. Gentamicin resistance gene was extracted from gel agarose by BioneerAccuPrep Gel Purification Kit. Quantity and quality of the purified extracted DNA were measured by Nanodrop (Thermo-scientific).

### Subcloning of *aacC1* gene into plasmid pmET32a

*β*-lactamase gene Amp*R* was excised from plasmid pmET32a by means of restriction enzyme BspHI. A formulation, including 100ng of the purified *aacC1* gene and 400ng of the linear pmET32a, was used to sub-clone the *aacC1* gene into pmET32a via TA cloning (TA-cloning vector kit, Thermo-scientific).

### PCR amplification of E-lysis gene

A fragment of E-lysis gene (with product size of 365 bps) was amplified with the following primer pair: Fwd. primer, CCTCTGGCGGTGATAATGG, and Rev. primer, TCCTGTTTGATGGTGGTTGAC.

### Restriction enzyme digestion of pmET32b

Restriction enzyme digestion of pmET32b was performed with two distinct sets of enzymes, namely, *“BsphI*, *XhoI”* and *“HindIII”*.

### pmET32b sequencing

A part of pmET32b (with product size of 1289 bps), comprised of *aacC1* gene and a portion of the backbone, was amplified using Fwd. CTAACTACGGCTACACTA and Rev. CCTATCTCGGTCTATTCT primers. The resulting amplicon was then sequenced. The complete sequence of pmET32b has been added to the gene bank, with the accession number of KF561236.

### Bacterial transformation

Transformation of *E*. *coli* DH5*α* and wild type O78:K80 was performed by treating the cells with a combination of CaCl2, NaCl, KCl, MgCl2 and MgSO4. This was followed by a heat shock to the cells. The transformed bacteria were incubated overnight at 28°*C* and 42°*C*, on Luria-Bertani agar plates containing 10 *μg/ml* gentamicin, in order to form single colonies. Colonies which grew in 28°*C* but not in 42°*C* and had both E-lysis and gentamicin resistance genes were preserved at *−*80°*C* with a 20% glycerol solution.

### Bacterial ghost construction

Controlled expression of gene E of phage *φ*X174 together with promoter PR was exploited to construct *E*. *coli* O78:K80 and *E*. *coli* DH5*α* BGs. For this, single colonies, containing pmET32b, were used to inoculate 250ml of broth LB (with 10 *μ*l/ml gentamicin). This culture was kept in a shaker incubator (28°*C* and 120 rpm) until OD of 0.3–0.4 (and in some cases, up to 0.8) at 600nm was achieved. Subsequently, the temperature and the shaker speed were increased to 42°*C* and 150 rpm, to initiate the induction of the E-lysis gene expression. The lysis process of bacteria was monitored via measuring OD600 value as well as counting the number of viable cells, at different time points. *E*. *coli* BGs were harvested 6 hours after the induction of E-lysis gene expression, washed at least three times with sterile PBS, lyophilized and then stored at *−*80°*C*.

### Scanning electron microscopy (SEM)

Cells were washed three times and fixed using 2.5% glutaraldehyde in PBS (pH = 7.4). Following that, samples were post-fixed with 1% aqueous osmium tetroxide and were then dehydrated with 30%, 50%, 70%, 90% and 100% ethanol. Subsequently, samples were critical-point-dried and coated with gold-palladium alloy. SEM imaging was performed with FEI Nova NanoSEM 450 machine.

### Safety dose of *E*. *coli* O78:K80 BGs

Ross 308 broiler chickens (Simorgh chicken farm, Khorasan Razavi, Iran) were maintained under standard lightening, feeding and vaccination program prescribed for broiler chickens in pens with wood shavings (chicken farm of veterinary medicine school, Ferdowsi University of Mashhad). Chickens were randomly allocated into different treatment groups in all experiments. *E*. *coli* BGs were counted using fuchsin staining. Safety dose of *E*. *coli* O78:K80 BGs was explored by injection of different doses of BGs (ranging from 10^11^ to 1013CFU) or PBS (as control) into the caudal abdominal region of 5-days-old Ross 308 chickens (4 groups, n = 7 per group). Chickens were then monitored for any possible clinical symptoms (mortality as well as having any signs of depression, ruffled feathers, not eating, diarrhea, low performance and respiratory distress signs including coughing and sneezing), daily for a period of 25 days, at the end of which, necroscopy was conducted. The air sac lesions were scored according to Kleven et al., 1972 [18] while the pericardial and perihepatic lesions were scored according to Charleston et al., 1998 [19].

The necroscopy scoring system for air sacs, heart and liver was as follows for all experiment:

- Score of 1: (A) cloudiness of air sacs, (B) excessive fluid in the pericardium, (C) definite fibrination on the surface of the liver.
- Score of 2: (A) air sacs membranes are thickened, (B) extensive fibrination in the pericardial cavity, (C) extensive fibrination, adhesions, liver swelling and necrosis.
- Score of 3: membranes meaty appearance, with accumulation of a cheesy exudates in one air sacs.
- Score of 4: lesions the same as score of 3 but in two or more air sacs.

### Experimental infection

Establishment of experimental infection was attempted by trying 4 different combinations of predisposing factors and wild type *E*. *coli* O78:K80 as well as 1 negative control group (n = 5 per group) on total number of 25 Ross 308 chickens as detailed in Table 1.

**Table 1:** Experimental infection treatment groups.

Group	*E*.*coli* O78:K80 dose	*E*.*coli* Administration Method	Dexamethasone (Injection)	IB30X (eye drop)
1	10^11^	Injection	N/A	N/A
2	10^11^	Injection	N/A	day 21
3	10^9^	Injection	N/A	day 21
4	10^11^ (100ml)	Inhalation	days 18 to 21 (1mg/kg)	day 21
5	0	N/A	N/A	N/A

### Production of *E*. *coli* O78:K80 killed vaccine

*E*. *coli* O78:K80 bacteria were inactivated using 3% Formaldehyde. Next, 10% ALK(SO4)2 was added as adjuvant, followed by incubation at 37°C for about 5 hours.

### In-Vivo efficacy of the *E*. *coli* O78:K80 BGs and killed vaccine

Total number of 85 chickens (5 groups, n = 17 per group) were used for this experiment. Chickens were immunized with either killed vaccine or *E*. *coli* O78:K80 BGs on days 7, 14 and 21. BG vaccinations were performed by injecting 1 ml (10^10^ BG/ml) of BGs at caudal abdominal area or spraying 100 ml of BGs (10^10^ BG/ml) into a closed chamber enclosing the chickens on days 7, 14 and 21. Treatment groups were as follows: (1) Challenged with wild type *E*. *coli* O78:K80 with no immunization; (2) Negative control with no immunization and no challenge; (3) Immunization with *E*. *coli* O78:K80 BGs (or killed vaccine) through subcutaneous injection and challenge with wild type *E*. *coli* O78:K80; and (4) Immunization with *E*. *coli* O78:K80 BGs through respiratory system and challenge with wild type *E*. *coli* O78:K80. Chickens were monitored for mortality as well as having any signs of respiratory distress (coughing and sneezing), depression, ruffled feathers, not eating, diarrhea and low performance daily after the challenge. Birds were sacrificed when showed any sign of discomfort. Decapitation was used with dedicated and clean equipment and it was ensured that the head is separated from the body completely and quickly by a person with technical proficiency.

### Sampling for evaluation of immune response and necropsy lesions

Serum samples were collected on days 14, 25 and 38 to evaluate IgG and IFN*γ*. Further, tracheal lavage for IgA evaluation and necroscopy of the carcasses were performed on days 25 and 38. IgY (A00165, GeneScript), IFN*γ* (CSB-PA15594B0Rb, CUSABIO) and IgA (A30-103P, Bethyl laboratories) HRP conjugates as well as O78:K80 BG, as the coating antigen, were utilized for conducting the indirect enzyme linked immunosorbent assay (ELISA).

### Statistical analysis

P-values for antibody titers were calculated using a two-tailed t-test. Necroscopy scores of the control and different treatment groups were compared using ANOVA as well as kruskal-wallis test via GraphPad Prism 5.0.

## Results

### Construction of bacterial ghost

As the first step to construct the BG of wild type *E*. *coli* O78:K80, the bacteria was transformed with E-lysis plasmid pmET32a, which had the ampicillin resistant *β*-lactamase gene as a marker. However, the wild type O78:K80 was observed to be resistant to ampicillin itself. This left no option but to replace *β*-lactamase with another antibiotic resistant gene to which, O78:K80 was not resistant.

**Fig 2:**
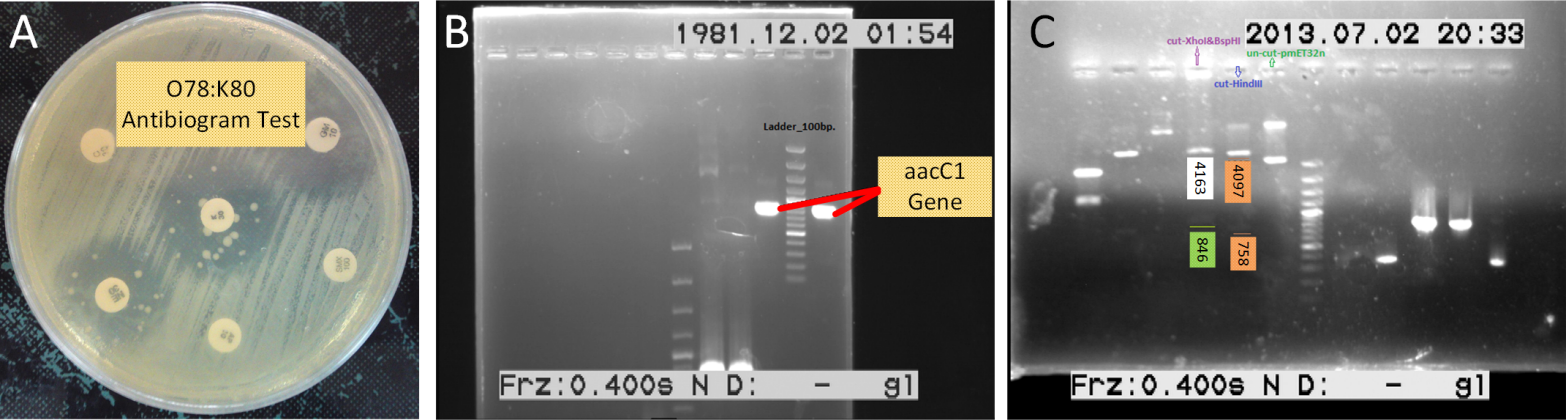
(A) The sensitivities of the wild type *E*. *coli* O78:K80 to ampicillin 10, Chloramphenicol 30, kanamycin 30, sulfametoxazol 100, gentamicin 10 and Neomycin 30 discs are shown; (B) amplification of gene *aacC1* from plasmid pCOM8. The two indicated bands are representative of *aacC1* gene amplified by PCR reaction. The ladder size is 100bp; (C) Gel electrophoresis of restriction enzyme digestion products for plasmid pmET32b: pmET32b was digested with two different sets of restriction enzymes *“Xhol*, *BspHI”* and *“HindIII”*. Products related to each set of digestion are shown. 1Kbp ladder was used.

In order to select a proper marker for pmET32a, on antibiogram test was performed on wild type O78:K80, using 6 different antibiotics. It was observed that, wild type O78:K80 was resistant to 5 out of 6 and in fact, gentamicin was the only antibiotic to which, O78:K80 was completely sensitive (Fig 2A). As a result of this finding, we proceeded with the gentamicin resistance gene, *aacC1*, as the replacement plasmid marker. Replacement process of Amp*R* with *aacC1* is schematically demonstrated in Fig 1. In a successful attempt, restriction enzyme BspHI was employed to excise *β-*lactamase gene out of the plasmid pmET32a. Also, PCR was performed to amplify gene *aacC1* from plasmid pCOM8 (Fig 2B). This was followed by purifying the PCR product and cloning aaC1 into plasmid pmET32a. The accuracy of the cloning was confirmed by digestion with two distinct sets of restriction enzymes, namely *“BsphI*, *XhoI”* and *“HindIII”*. Each set produced 2 bands with 846 and 4163 bps for the former and 758 and 4097 bps for the latter (Fig 2C). This was consistent with the *in silico* mapping of the digestion.

A fragment of the plasmid, comprised of a portion of the cloned gene and a part of the plasmid backbone, was amplified by Fwd, CTAACTACGGCTACACTA and Rev, CCTATCTCGGTCTATTCT primers and the sequence was confirmed by Sanger sequencing technique (S1 Fig). The resulting construct, pmET32b, was submitted to GenBank under accession number of KF561236 [20].

Wild type *E*. *coli* O78:K80 and DH5*α* were successfully transformed with plasmid pmET32b. The successful attempt of amplifying Gentamicin resistance and E-lysis genes confirmed that the transformed colonies possessed the plasmid. The transformed colonies were also incubated in both 28°*C* and 42°*C* to examine the functionality of E-lysis product. Colonies which grew at 28°*C* but not at 42°*C* (S2 Fig) and had both E-lysis and gentamicin resistance genes were considered as successfully transformed bacteria. They were then preserved at *−*80°*C* in 20% glycerol.

**Fig 3:**
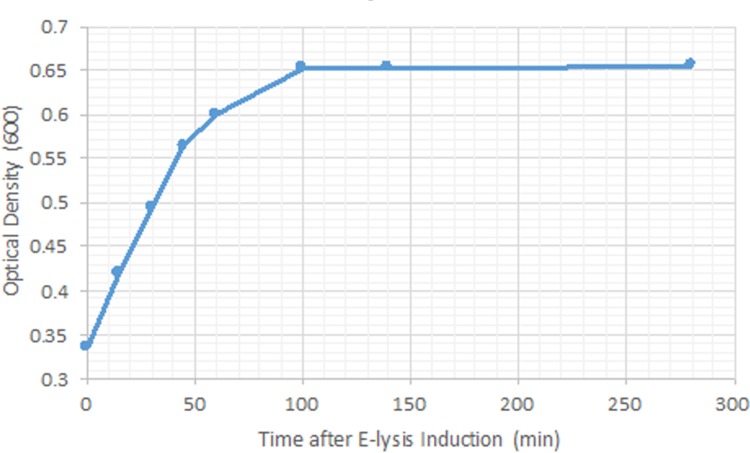
Trend of OD during E-lysis process.

**Fig 4:**
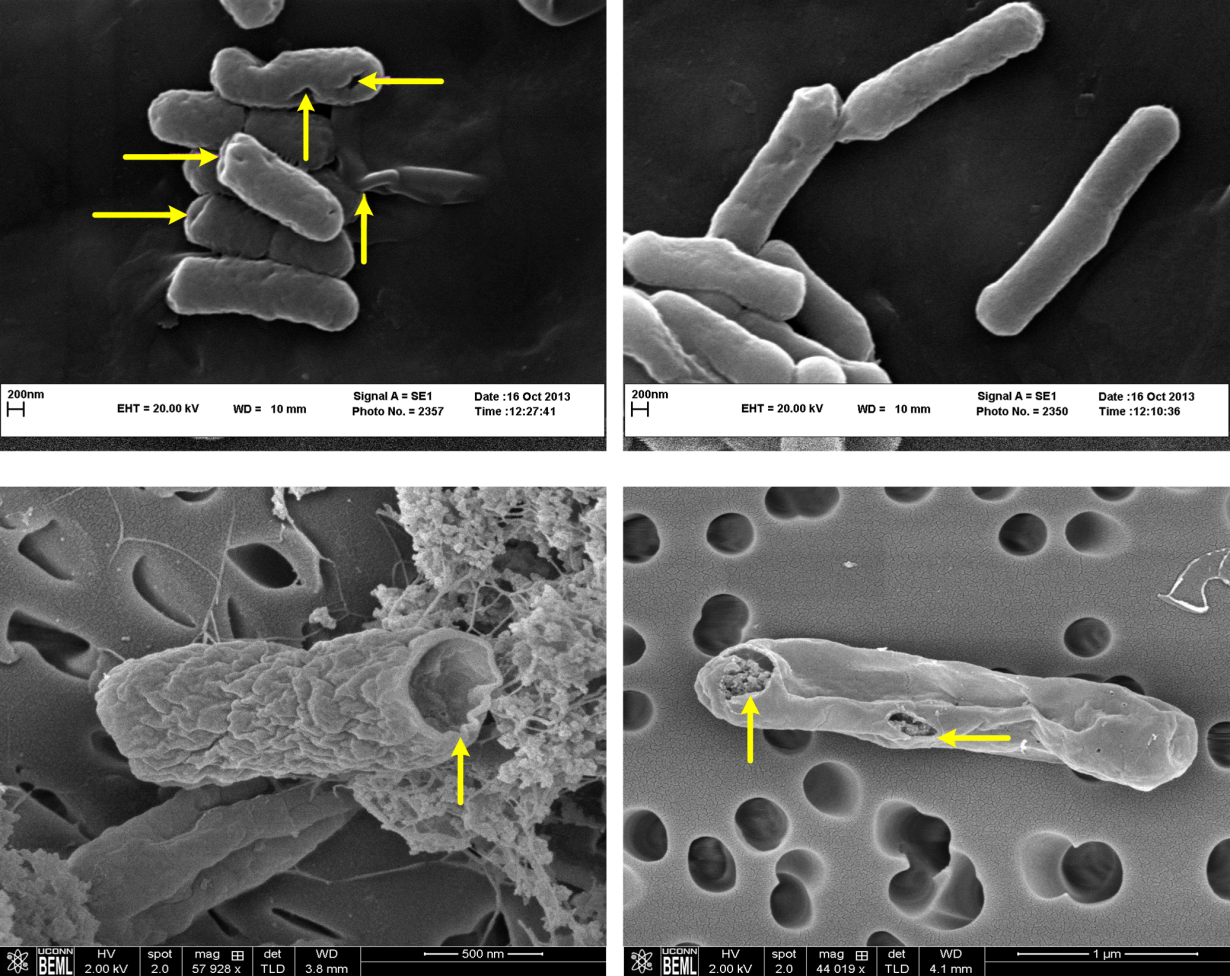
Assessment of *E*. *coli* O78:K80 and DH5*α* ghosts using SEM: (A) Left shows pathogenic *E*. *coli* O78:K80 ghosts and right depicts the wild type *E*. *coli* O78:K80; (B) *E*. *coli* DH5*α* ghosts. Transmembrane lysis tunnels are shown by arrows.

We took advantage of the temperature inducibility of E-lysis promoter to initiate E-lysis protein expression with increasing the temperature from 28°*C* to 42°*C*, which in turn resulted in pore formation on the cell wall of the bacteria and production of ghosts. Ghost formation was investigated using three different methods. First, colony forming units (CFU) were counted with sampling interval of 30 minutes, following the E-lysis induction. At +1 hour (E-lysis induction defines the reference for time), the number of CFUs started to decrease and at +3 hours, no detectable CFUs were observed. Next, the OD of the bacterial culture was measured with intervals of 15 minutes, following E-lysis induction (Fig 3). The measured OD for O78:K80 shows a rapid growth with a constant slope up to around +45 minutes and then the slope starts to decline until it reaches 0 at around +100 minutes and from there on, the OD remains at the constant value of 0.65 for the rest of the process (Fig 3). Finally, SEM was used to investigate the formation of pores on the cell wall of both O78:K80 and DH5*α* bacteria. Pores were observed in bacterial cell dividing regions as well as the poles (Fig 4).

### Experimental infection and safety dose of O78:K80 BG vaccine

Experimental infection was established by optimizing two parameters namely, dose and method of administration. Doses ranged from 109 to 1013 CFUs and administration was conducted via injection as well as through the respiratory system into 21-day-old Ross 308 broiler chickens. In order to apply predisposing factors, administration of several combinations of two components, namely 1–2 mg/kg dose of Dexamethasone and 30x dose of infectious bronchitis H120 eye drop vaccine (IB30x), were utilized. Infection in chicken carcasses was evaluated and scored [19, 21]. According to the necroscopy scores, injection of 1011 CFUs of *E*. *coli* O78:K80 together with IB30x yielded the highest score, yet without mortality. Also, the bacteria were recoverable from heart, liver and airsacs of the infected chickens. This treatment was chosen to establish experimental infection for the subsequent experiments.

Doses in the range of 10^9^ to 10^13^ O78:K80 BGs were tested on 5-days-old chickens to determine the safety dose of the O78:K80 BG vaccine. Chickens were euthanized on day 26 and the infection symptoms were scored. None of the chickens showed any adverse effect. Hence, no significant lesion was observed in the treatment versus the control groups, ensuring the safety of the selected doses. Since a wide window of safety dose was preferred, the dose of 1010 O78:K80 BGs was chosen for the forthcoming immunizations to ensure the safety of the birds during vaccination (even 10^3^ times more BGs than the vaccination dose, would still be safe for chickens). It is also worth noting that usually doses equal or less than 10^9^ are used for *E*. *coli* vaccination [22]. Therefore, 10^10^ O78:K80 BGs was considered a safe reasonable vaccination dose.

### Efficacy of O78:K80 BG-based vaccine *in-Vivo*

In order to test the efficacy of O78:K80 BG vaccine *in-vivo*, Ross 308 broiler chickens were immunized with three doses of 10^10^ O78:K80 BGs or killed vaccine (on days 7, 14 and 22). This was followed by challenging the chickens with wild type O78:K80 on day 33.

**Fig 5:**
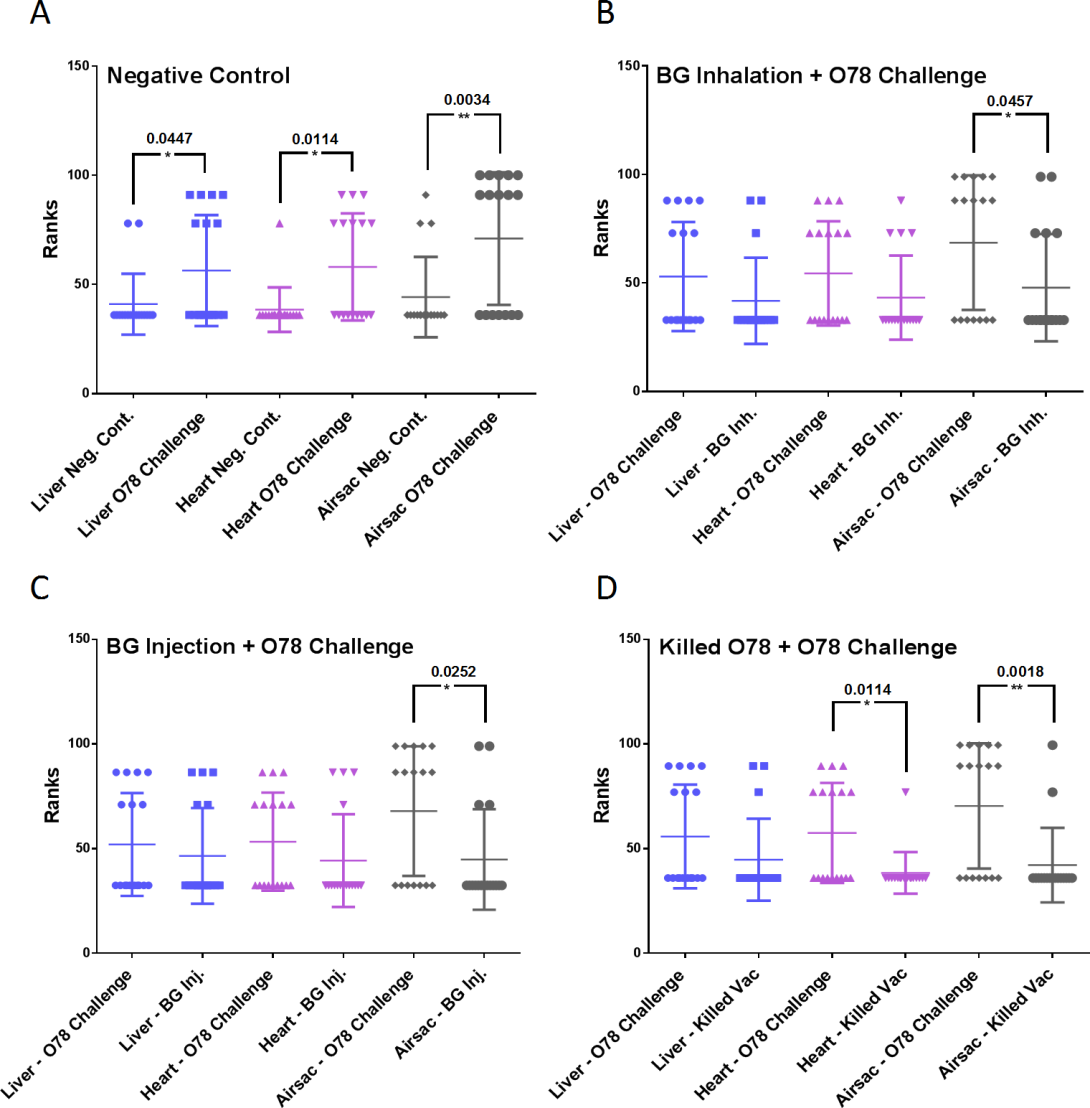
Chickens were immunized with killed or O78:K80 BG vaccine and were challenged with wild type O78:K80 *E*. *coli* bacteria. Necroscopy evaluations were performed on chickens’ carcasses as described in the Methods section. All graphs represent the comparison of Lesions in chickens challenged with wild type O78:K80 *E*. *coli* bacteria (without any immunizations) and chickens that were immunized with PBS (negative control), BG or killed vaccines. P-values are shown as indicated.

Necroscopy evaluation and lesions scoring were conducted on days 26 and 38. The difference in lesion scores between the group challenged with wild type *E*. *coli* O78:K80 and the negative control group was statistically significant for all major organs (liver, heart and air sacs) which shows a successful experimental infection of Colibacillosis (Fig 5A). Compared to the positive control group, a significant reduction was observed in the scores associated with air sacs lesions in the group that was immunized by *E*. *coli* O78:K80 BG vaccine, via injection as well as inhalation. Immunized chickens with *E*. *coli* O78:K80 BGs also had reduced lesions in liver and heart, although the difference was not statistically significant (Fig 5B and 5C). While immunization with killed vaccine reduced lesion scores both in the heart as well as the air sacs (Fig 5D). However, no significant difference was observed between immunization with BG-based and killed vaccines which indicates that *E*. *coli* O78:K80 BGs vaccination gives a comparable protection level as a killed *E*. *coli* O78:K80 vaccine plus adjuvant.

**Fig 6:**
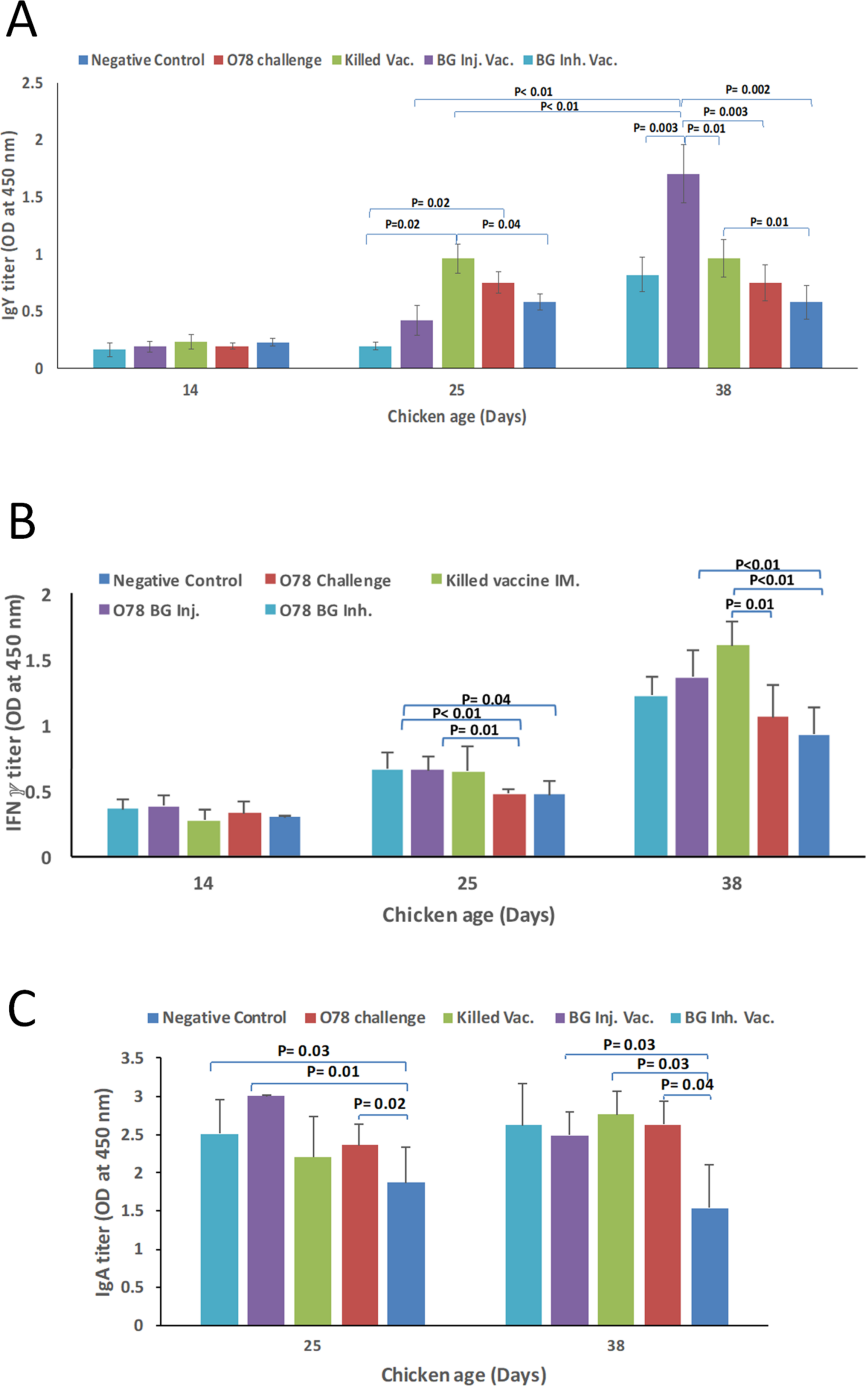
(A), (B) IgY and IFN*γ* titers on serum sample of chickens immunized with different vaccine types on days 14, 25 and 38; (C) IgA titer in tracheal lavage samples from different groups of immunized chickens on days 25 and 38. P-values are shown as indicated.

The amounts of systemic IgY in serum samples of different experimental groups were evaluated. The titer of the IgY, on day 14, showed no difference among different groups. However, on day 25, IgY was significantly higher in the group immunized with killed O78:K80 compared to the group immunized with BG vaccine through inhalation and the negative control group. Interestingly, on day 38, the amount of IgY antibody in BG vaccinated groups showed more than 2-fold increase with respect to day 25. Further, these values were significantly higher than that of the group immunized with killed O78:K80. Although IgY content increased with age in the negative control group, the difference was not statistically significant (Fig 6A).

Tracheal lavage samples on days 25 and 38 were used to evaluate the amount of IgA antibody. The content of IgA antibody was significantly higher, both in the group that was vaccinated with BG via injection, and the group that was vaccinated with BG through inhalation (but not the group that was immunized with killed O78:K80), as compared to the negative control group. However, the content of IgA antibody on day 38, was slightly higher in the group that was immunized by killed O78:K80, relative to the group that was vaccinated with BG (Fig 6C). The two groups that were vaccinated with BG (but not the group that was immunized with killed O78:K80) showed significantly higher amount of IFN*γ*, on day 25, with respect to the group that was only challenged with wild type *E*. *coli* O78:K80. However, compared to the negative control group, the amount of this cytokine was significantly higher only in the group that was immunized with BG via injection as well as the group that was immunized with killed vaccine (Fig 6B).

## Discussion

The devastating impacts of avian Colibacillosis are particularly evident in the poultry farms of developing countries, which due to poor bio-security systems are extremely prone to this disease. To design a vaccine against Colibacillosis, one should consider that, most of the APECs are resistant to several antibiotics [5, 23]. Therefore, it is critical for the vaccine designer to be cautious about spreading the antibiotics resistance gene in the environment.

In this study, we attempted to address this challenge by taking advantage of the BG technology. The benefit of using bacterial ghost technology is that it can eliminate the risk of genetic spread, by discharging the cellular content through the pores that are formed on the cell wall and/or by expression of staphylococcal nuclease (SNUC) to degrade DNA molecules inside the BG [24]. More particularly, we successfully produced a homologous BG-based vaccine against pathogenic *E*. *coli* O78:K80 which is among the three most important bacterial serotypes causing Colibacillosis (the other two are O1 and O2 [25, 26]). The composition of the ions used for O78:K80 transformation had to be somewhat more complex than the ones used for *E*. *coli* DH5*α*, which was probably due to the thicker cell wall of the wild type *E*. *coli* O78:K80. The resulting BG-based vaccine was able to stimulate the immune response and protect chickens against experimental infection of Colibacillosis to some extent. It was observed that administration of *E*. *coli* O78:K80 BGs via injection was more effective than inhalation. No significant difference was observed between the immunized versus the control groups on day 14, which probably was due to the fact that immunization dose was not enough to elicit a response peak in as short as 7 days. However, the results of titrations conducted on day 28 displayed significantly more immunoglobulin content in the vaccinated groups versus the negative control group.

Since all pathogenic epitopes on bacterial cell wall remain intact during the BG production process, BGs mimic live vaccines and are capable of stimulating humoral and cellular immune responses [12] which, according to Sadeyen et. al, 2014 [27], are both required to battle Colibacillosis. Therefore, BGs are expected to provide a more effective treatment with respect to the killed vaccine. Our results showed that the protective effects of BG-based vaccine were comparable to the killed vaccine. The fact that the killed vaccine was administered in combination with adjuvant, while BG-based vaccine was administered without it emphasizes the adjuvanticity inherent in the BG vaccines. On the other hand, as BGs are incapable of proliferation, they provide a safer alternative relative to live vaccines such as the O2 vaccine, which showed promising efficacy in turkeys [28], as there is always a concern that even attenuated bacteria may convert back to wild type. Furthermore, as the cellular content of the bacteria will be washed out, the risk of the antibiotic resistance gene spreading is eliminated. A commercially available live vaccine, Poulvac by Pfizer, has shown to be safe and partially efficient in broiler chickens [29]. Although, the cost and labor associated with the production of live attenuated vaccines, in reality, make vaccine customization, using this method, impractical. Conversely, the simplicity of the BG-based vaccine advocates highly customizable solutions which is key to finding treatments for Avian Colibacillosis in different countries with different environmental factors. Exploring treatments for Colibacillosis, one will find several other types of vaccines that have not made it to the commercialization stage. These include ultrasonic inactivated *E*. *coli* O2:K1 [30] and recombinant for Colibacillosis increased serum survival gene iss [31], to name a few.

Other than vaccination, other non-antibiotic approaches have also been attempted for treatment of Colibacillosis. One example is oral administration of Bacillus subtilis bacterial spores which outcompetes *E*. *coli* and suppresses its proliferation in chickens’ intestine [32]. Nevertheless, as spores dissipate in time, *E*. *coli* will eventually replace them again. Another example is usage of anti-*E*. *coli* phages which, is currently practiced in hospitals for infant diarrhea. However, there is some concern about the safety of this method [33].

It looks like, in spite of all the hurdles associated with making vaccines for Colibacillosis, mainly due to the multi-cause nature of this disease, vaccination surpasses other existing approaches in terms of efficacy, safety and cost.

Production of wild type *E*. *coli* O78:K80 BGs, as established in this work, sets the ground for taking the next step, namely, designing multivalent vaccines which are deemed to have higher efficacy. Furthermore, simplicity and cost effectiveness of BG-based strategy for making vaccines has enabled production of customized (i.e., autogenous) vaccine products against Colibacillosis to be used by individual poultry fields. This is particularly important as one serotype may show different properties when isolated from poultry farms in different geographical locations [34].

## Supporting information

S1 Fig pmET32b sequencing. (TIF)

S2 Fig Temperature dependent induction of E-lysis protein expression. (TIF)

S1 Table NC3Rs ARRIVE guidelines checklist. (PDF)
