# Supplementary figures and images for: Bacterial ghost of avian pathogenic E. coli (APEC) serotype O78:K80 as a homologous vaccine against avian colibacillosis
Source: PLoS One. 2018 Mar 22;13(3):e0194888. doi: 10.1371/journal.pone.0194888 (PMC5864078; doi:10.1371/journal.pone.0194888)

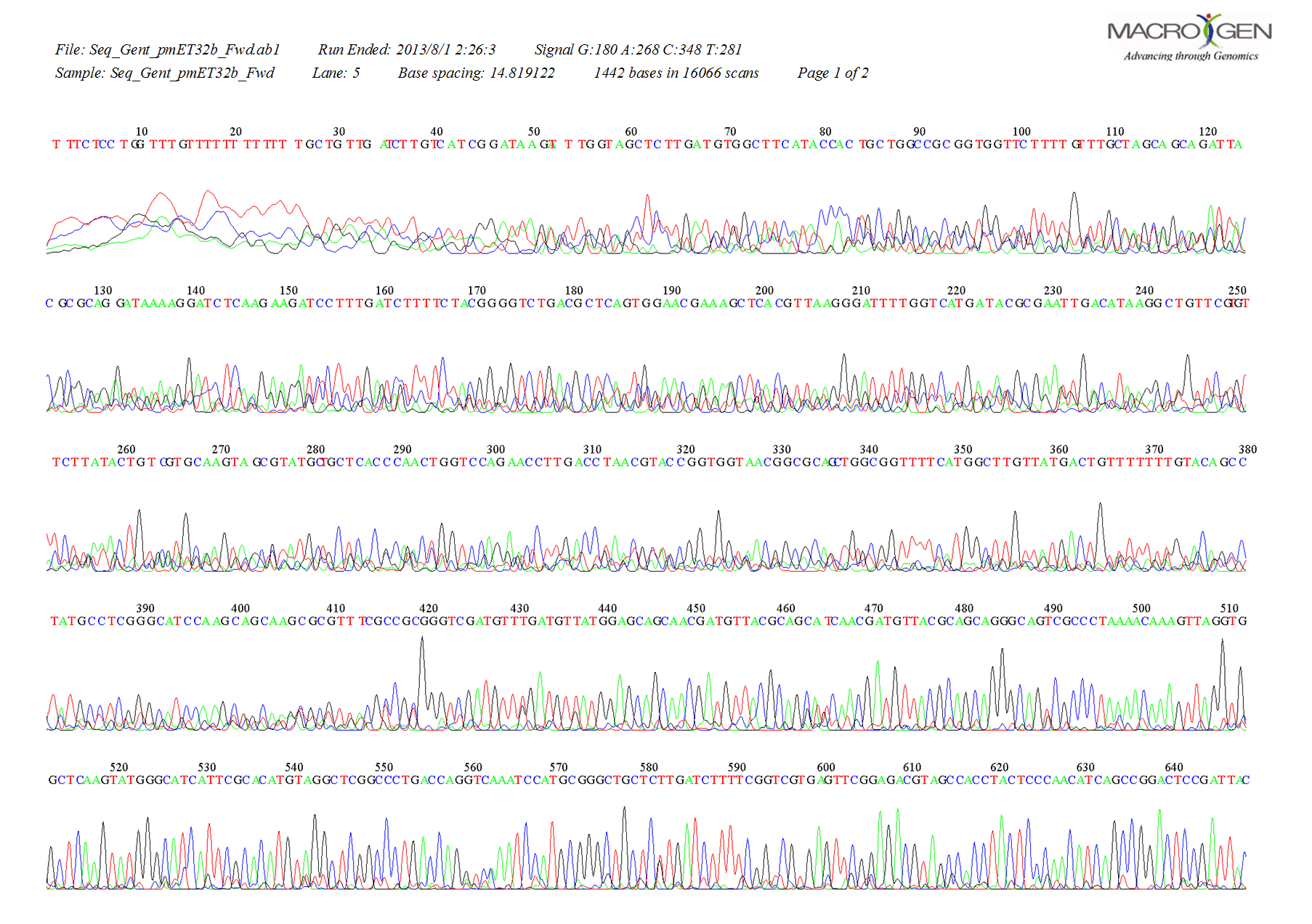

Supplement: S1 Fig — (TIF) [file pone.0194888.s001.tif]

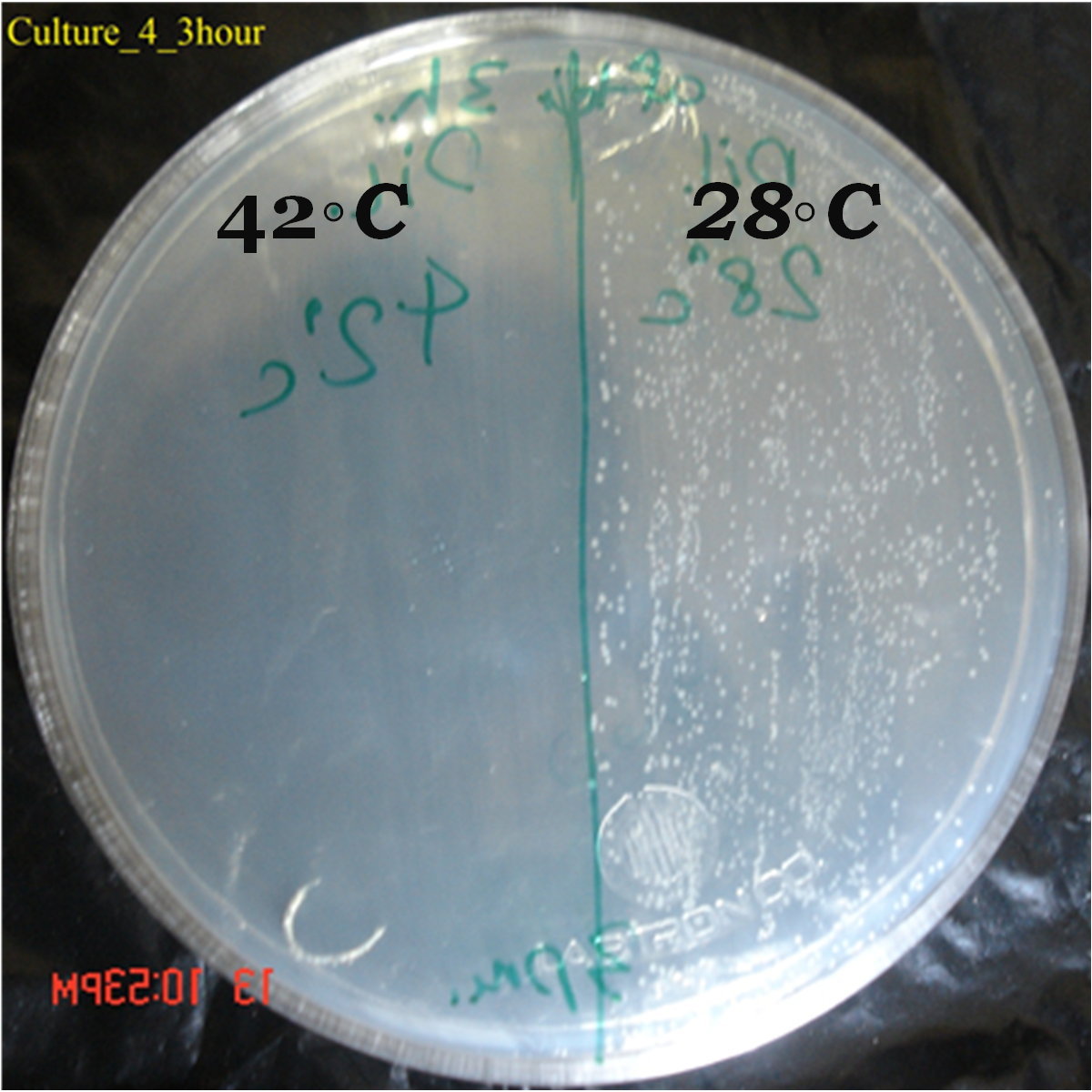

Supplement: S2 Fig — (TIF) [file pone.0194888.s002.tif]
